# Complete genome sequence of *Halomicrobium mukohataei* type strain (arg-2^T^)

**DOI:** 10.4056/sigs.42644

**Published:** 2009-11-22

**Authors:** Brian J. Tindall, Susanne Schneider, Alla Lapidus, Alex Copeland, Tijana Glavina Del Rio, Matt Nolan, Susan Lucas, Feng Chen, Hope Tice, Jan-Fang Cheng, Elizabeth Saunders, David Bruce, Lynne Goodwin, Sam Pitluck, Natalia Mikhailova, Amrita Pati, Natalia Ivanova, Konstantinos Mavrommatis, Amy Chen, Krishna Palaniappan, Patrick Chain, Miriam Land, Loren Hauser, Yun-Juan Chang, Cynthia D. Jeffries, Thomas Brettin, Cliff Han, Manfred Rohde, Markus Göker, Jim Bristow, Jonathan A. Eisen, Victor Markowitz, Philip Hugenholtz, Hans-Peter Klenk, Nikos C. Kyrpides, John C. Detter

**Affiliations:** 1 DSMZ - German Collection of Microorganisms and Cell Cultures GmbH, Braunschweig, Germany; 2 DOE Joint Genome Institute, Walnut Creek, California, USA; 3 Los Alamos National Laboratory, Bioscience Division, Los Alamos, New Mexico, USA; 4 Biological Data Management and Technology Center, Lawrence Berkeley National Laboratory, Berkeley, California, USA; 5 Lawrence Livermore National Laboratory, Livermore, California, USA; 6 Oak Ridge National Laboratory, Oak Ridge, Tennessee, USA; 7 HZI - Helmholtz Centre for Infection Research, Braunschweig, Germany; 8 University of California Davis Genome Center, Davis, California, USA

**Keywords:** extreme halophile, mesophile, free-living, motile, non-pathogenic, facultatively anaerobic, rod-shaped, *Halobacteriaceae*

## Abstract

*Halomicrobium mukohataei* (Ihara *et al*. 1997) Oren *et al.* 2002 is the type species of the genus *Halomicrobium*. It is of phylogenetic interest because of its isolated location within the large euryarchaeal family *Halobacteriaceae. H. mukohataei* is an extreme halophile that grows essentially aerobically, but can also grow anaerobically under a change of morphology and with nitrate as electron acceptor. The strain, whose genome is described in this report, is a free-living, motile, Gram-negative euryarchaeon, originally isolated from Salinas Grandes in Jujuy, Andes highlands, Argentina. Its genome contains three genes for the 16S rRNA that differ from each other by up to 9%. Here we describe the features of this organism, together with the complete genome sequence and annotation. This is the first completed genome sequence from the poorly populated genus *Halomicrobium*, and the 3,332,349 bp long genome (chromosome and one plasmid) with its 3416 protein-coding and 56 RNA genes is part of the *** G****enomic* *** E****ncyclopedia of* *** B****acteria and* *** A****rchaea * project.

## Introduction

Strain arg-2^T^ (= DSM 12286 = ATCC 700874 = JCM 9738) is the type strain of the species *Halomicrobium mukohataei*, and represents the type species of the genus *Halomicrobium* [1]. *H. mukohataei* was initially described as *Haloarcula mukohataei* (basonym) by Ihara *et al.* 1997 [2]. *H. mukohataei* is a motile, extremely halophilic euryarchaeon. The organism is of significant interest for its isolated position in the tree of life within the genus *Halomicrobium* in the family *Halobacteriaceae*. *H. katesii* [3] is currently the only other cultivated member of the genus *Halomicrobium.* Only two uncultivated archaeal clones related to the genus (>98% sequence identity) have been reported from diversity screenings: clone XCDLW-A62 from saline lakes on the Tibetan Plateau (FJ155620), and clone SA93 from an athalassohaline environment in the Tirez Lagoon in Spain (EU722674). No phylotypes from environmental samples or genomic surveys could be directly linked to *H. mukohataei.*  Here we present a summary classification and a set of features for *H. mukohataei* arg-2^T^, together with the description of the complete genomic sequencing and annotation.

### Classification and features

**Figure 1:**
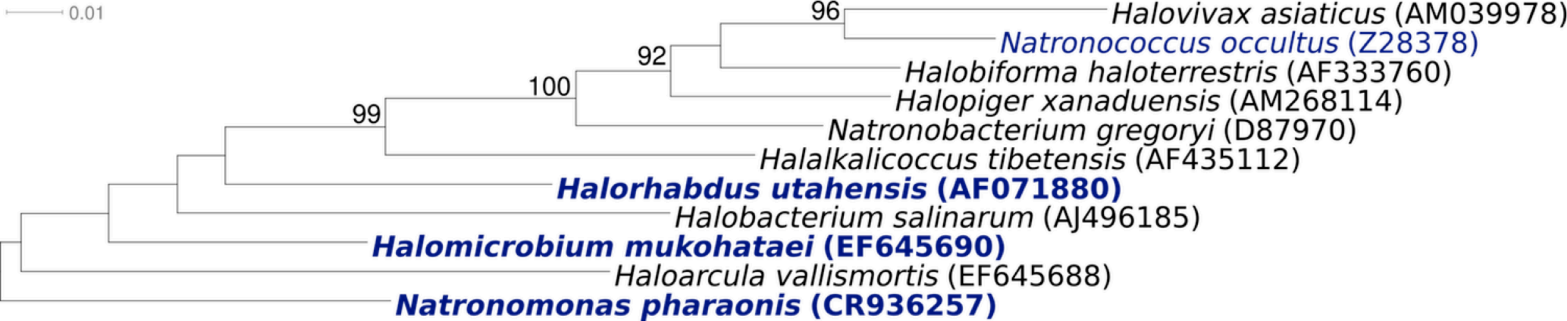
Phylogenetic tree of *H. mukohataei* arg-2^T^, all type strains of the genera *Halomicrobium* and *Haloarcula* and type strains of other selected members of the family *Halobacteriaceae*, inferred from 1,430 aligned characters [4,5] of the 16S rRNA gene using the neighbor-joining algorithm and K2P distances [6]. The tree was rooted with *Natronomonas pharaonis,* the deepest branching member of the family *Halobacteriaceae*. The branches are scaled in terms of the expected number of substitutions per site. Numbers above branches are support values from 1,000 bootstrap replicates if larger than 60%. Strains with a genome sequencing project registered in GOLD [7] are printed in blue; published genomes in bold, e.g. the GEBA genome from *Halorhabdus utahensis* [8].

Figure 1 shows the phylogenetic neighborhood of *H. mukohataei* strain arg-2^T^ in a 16S rRNA based tree. Two of the three 16S rRNA gene copies in the *H. mukohataei* arg-2^T^ genome are identical, but differ by 131 nucleotides (9%) from the third copy (23S rRNA gene sequences differ by only 1-1.7%, this study). Studies on the ribosomes indicate that operons which differ significantly in their sequence are expressed under different environmental conditions [9], as has also been reported for members of the genus *Haloarcula* [10]. The symbols rrnA and rrnB used in Figure 1 for these distinct rRNA copies in *Haloarcula* and *Halomicrobium* are in accordance with the designations used by Cui *et al*. 2009 [9]. The two identical 16S rRNA genes differ in one nucleotide from the previously reported reference sequence of strain arg-2^T^ derived from JCM 9738 (EF645690).

**Figure 2:**
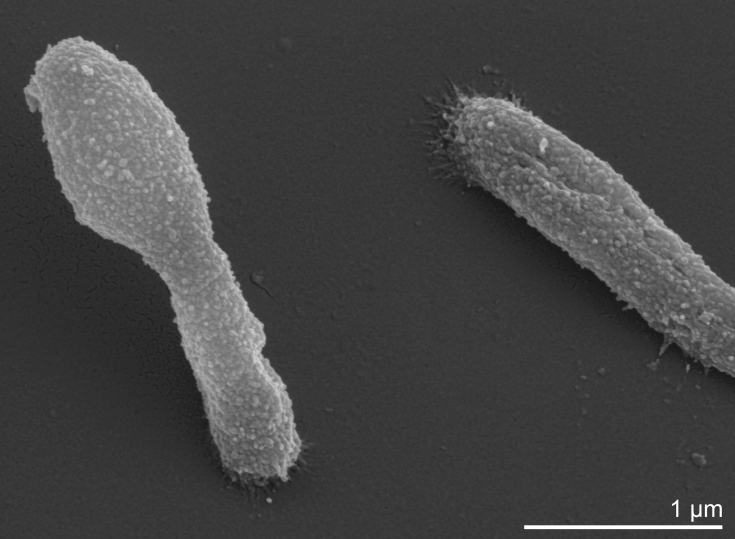
Scanning electron micrograph of *H. mukohataei* arg-2^T^

*H. mukohataei* is rod shaped (Table 1), but may produce pleomorphic cells in the stationary phase [1] (Figure 2). There are conflicting reports concerning the type of flagellation, which may be either polar or in tufts or peritrichous [1]. Gas vacuoles have not been reported and resting stages such as spores are not produced. Cells are Gram-negative, although peptidoglycan is probably absent [1]. Strain arg-2^T^ grows under aerobic conditions, but may also grow anaerobically in the presence of nitrate [1]. Arginine does no support anaerobic growth. Acids are produced from glucose, galactose, mannose, ribose, sucrose, maltose and glycerol [1]. Glucose, galactose, sucrose, maltose and glycerol support growth as single carbon and energy sources. Starch is hydrolyzed [1], however, gelatin, casein and Tween 80 are not hydrolyzed. Requires at least 2M NaCl to maintain cell shape, with optimal growth occurring at 3.0-3.5 M NaCl. Catalase and oxidase positive. Optimal growth temperature is 40-45°C [1].

**Table 1:** Classification and general features of *H. mukohataei* arg-2^T^ in accordance to the MIGS recommendations [11]

**MIGS ID**	**Property**	**Term**	**Evidence code**
	Current classification	Domain *Archaea*	TAS [12]
		Phylum *Euryarchaeota*	TAS [13]
		Class *Halobacteria*	TAS [14]
		Order *Halobacteriales*	TAS [15]
		Family *Halobacteriaceae*	TAS [16]
		Genus *Halomicrobium*	TAS [1]
		Species *Halomicrobium mukohataei*	TAS [1]
		Type strain arg-2	TAS [1]
	Gram stain	negative	TAS [1]
	Cell shape	short rod with variable cell length; above 45°C spherical morphology	TAS [1]
	Motility	motile, multiple peritrichous or tufts of flagella	TAS [1]
	Sporulation	non-sporulating	NAS
	Temperature range	mesophile, <52°C	TAS [1]
	Optimum temperature	40-45°C	TAS [1]
	Salinity	extremely halophilic; requires 2.5-4.5 M NaCl, optimum 3-3.5 M NaCl	TAS [1]
MIGS-22	Oxygen requirement	essentially aerobic; grows anaerobically with nitrate as electron acceptor	TAS [1]
	Carbon source	glucose, galactose, sucrose, maltose, glycerol	TAS [1]
	Energy source	glucose, galactose, sucrose, maltose, glycerol	TAS [1]
MIGS-6	Habitat	soils of salt flats	TAS [2]
MIGS-15	Biotic relationship	Free living	NAS
MIGS-14	Pathogenicity	none	TAS [17]
	Biosafety level	1	TAS [17]
	Isolation	soils of salt flats in Salinas Grandes from Andes highlands	TAS [1]
MIGS-4	Geographic location	Jujuy, Argentina	TAS [1]
MIGS-5	Sample collection time	1991	TAS [1]
MIGS-4.1 MIGS-4.2	Latitude, Longitude	-22.66, -66.23	NAS
MIGS-4.3	Depth	not reported	
MIGS-4.4	Altitude	Sea level	NAS

Evidence codes - IDA: Inferred from Direct Assay (first time in publication); TAS: Traceable Author Statement (i.e., a direct report exists in the literature); NAS: Non-traceable Author Statement (i.e., not directly observed for the living, isolated sample, but based on a generally accepted property for the species, or anecdotal evidence). These evidence codes are from the Gene Ontology project [18]. If the evidence code is IDA, then the property was directly observed for a living isolate by one of the authors or an expert mentioned in the acknowledgements.

### Chemotaxonomy

The quinone composition of *H. mukohataei* arg-2^T^ has not been investigated, but based on reports from other members of the family *Halobacteriaceae* menaquinones with eight isoprenoid units are likely to be present. Typically both MK-8 and MK-8 (VIII-H_2_) may be predicted. The lipids are based on diphytanyl ether lipids. The major phospholipids are the diphytanyl ether analogues of phosphatidylglycerol and methyl-phosphatidylglycerophosphate (typical of all members of the family *Halobacteriaceae*), the diether analogue of phosphatidylglycerol sulfate is present [1]. Glycolipids have been reported, one of which has a molecular weight typical of a sulfate diglycosyl diphytanyl ether, the structure of which has not been determined [1]. A diglycosyl diphytanyl ether lipid is also present. The pigments responsible for the red color of the cells have not been recorded, but it may be predicted that they are carotenoids, probably bacterioruberins. Outer cell layers are probably proteinaceous. The presence of peptidoglycan has not been investigated, but is generally absent from members of this family *Halobacteriaceae.*

## Genome sequencing and annotation

### Genome project history

This organism was selected for sequencing on the basis of its phylogenetic position, and is part of the *** G****enomic* *** E****ncyclopedia of* *** B****acteria and* *** A****rchaea * project. The genome project is deposited in the Genome OnLine Database [7] and the complete genome sequence in GenBank Sequencing, finishing and annotation was performed by the DOE Joint Genome Institute (JGI). A summary of the project information is shown in Table 2.

**Table 2:** Genome sequencing project information

**MIGS ID**	**Property**	**Term**
MIGS-31	Finishing quality	Finished
MIGS-28	Libraries used	Three genomic libraries: two Sanger libraries - 8 kb pMCL200 and fosmid pcc1Fos and one 454 pyrosequencing standard library
MIGS-29	Sequencing platforms	ABI3730, 454 GS FLX
MIGS-31.2	Sequencing coverage	13.4x Sanger; 31× pyrosequencing
MIGS-30	Assemblers	Newbler version 1.1.02.15, phrap
MIGS-32	Gene calling method	Prodigal, GenePRIMP
	INSDC ID	CP001688
	Genbank Date of Release	September 9, 2009
	GOLD ID	Gc01100
	NCBI project ID	27945
	Database: IMG-GEBA	2501416928
MIGS-13	Source material identifier	DSM 12286
	Project relevance	Tree of Life, GEBA

### Growth conditions and DNA isolation

*H. mukohataei* arg-2^T^, DSM 12286, was grown in DSMZ medium 372 (Halobacterial Medium) [19] at 35°C. DNA was isolated from 1-1.5 g of cell paste using Qiagen Genomic 500 DNA Kit (Qiagen, Hilden, Germany) with a modified protocol for cell lysis, (procedure L), according to Wu *et al*. [20].

### Genome sequencing and assembly

The genome was sequenced using a combination of Sanger and 454 sequencing platforms. All general aspects of library construction and sequencing performed at the JGI can be found at the JGI website (http://www.jgi.doe.gov/). 454 Pyrosequencing reads were assembled using the Newbler assembler version 1.1.02.15 (Roche). Large Newbler contigs were broken into 3,703 overlapping fragments of 1,000 bp and entered into assembly as pseudo-reads. The sequences were assigned quality scores based on Newbler consensus q-scores with modifications to account for overlap redundancy and adjust inflated q-scores. A hybrid 454/Sanger assembly was made using the parallel phrap assembler (High Performance Software, LLC). Possible mis-assemblies were corrected with Dupfinisher or transposon bombing of bridging clones [21]. A total of 39 Sanger finishing reads were produced to close gaps, to resolve repetitive regions, and to raise the quality of the finished sequence. The error rate of the completed genome sequence is less than 1 in 100,000. Together, the combination of the Sanger and 454 sequencing platforms provided 44.4× coverage of the genome. The final assembly contains 48,917 Sanger reads and 443,713 pyrosequencing reads.

### Genome annotation

Genes were identified using Prodigal [22] as part of the Oak Ridge National Laboratory genome annotation pipeline, followed by a round of manual curation using the JGI GenePRIMP pipeline (http://geneprimp.jgi-psf.org/) [23]. The predicted CDSs were translated and used to search the National Center for Biotechnology Information (NCBI) nonredundant database, UniProt, TIGRFam, Pfam, PRIAM, KEGG, COG, and InterPro databases. Additional gene prediction analysis and functional annotation was performed within the Integrated Microbial Genomes Expert Review platform (http://img.jgi.doe.gov/er) [24].

## Genome properties

The genome is 3,332,349 bp long and comprises one main circular chromosome of 3.11 Mbp and one 219 kbp megaplasmid with a 65.5% GC content (Table 3, Figure 3a and Figure 3b). Of the 3,472 genes predicted, 3,416 were protein coding genes, and 56 RNAs. In addition, 66 pseudogenes were identified. The majority of the genes (59.4%) were assigned with a putative function while those remaining were annotated as hypothetical proteins. The properties and the statistics of the genome are summarized in Table 3. The distribution of genes into COGs functional categories is presented in Table 4.

**Table 3:** Genome Statistics

**Attribute**	**Value**	**% of Total**
Genome size (bp)	3,332,349	100.00%
DNA coding region (bp)	2,927,602	87.85%
DNA G+C content (bp)	2,183,712	65.53%
Number of replicons	2	
Extrachromosomal elements	1	
Total genes	3,472	100.00%
RNA genes	56	1.61%
rRNA operons	3	
Protein-coding genes	3,416	98.30%
Pseudo genes	66	1.90%
Genes with function prediction	2,081	59.88%
Genes in paralog clusters	610	17.55%
Genes assigned to COGs	2,135	61.44%
Genes assigned Pfam domains	2,079	59.83%
Genes with signal peptides	465	13.38%
Genes with transmembrane helices	874	25.15%
CRISPR repeats	2	

**Figure 3a:**
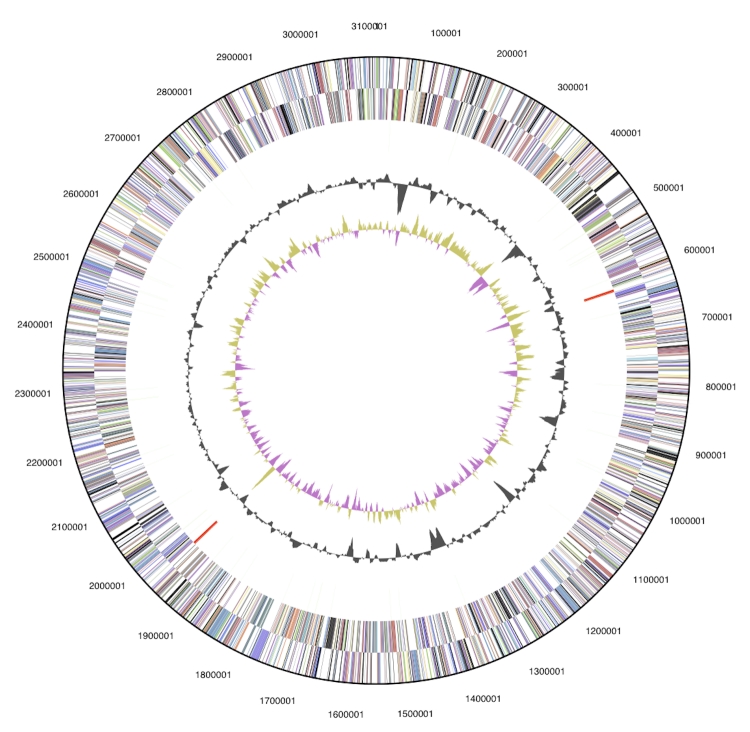
Graphical circular map of the chromosome. From outside to the center: Genes on forward strand (color by COG categories), Genes on reverse strand (color by COG categories), RNA genes (tRNAs green, rRNAs red, other RNAs black), GC content, GC skew.

**Figure 3b:**
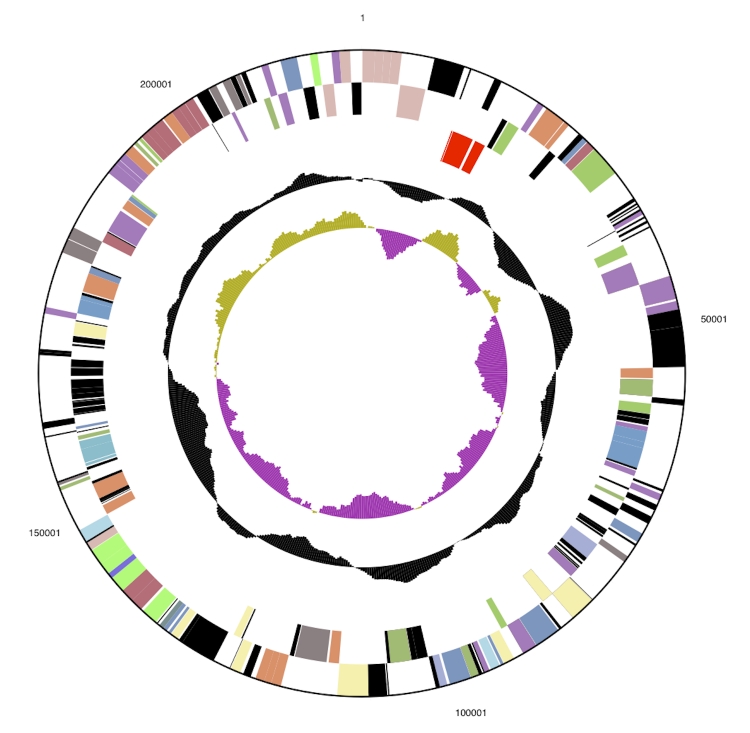
5.5x enlarged (vs. chromosome) graphical circular map of the megaplasmid.

**Table 4:** Number of genes associated with the general COG functional categories

**Code**	**Value**	**% age**	**Description**
J	157	4.6	Translation, ribosomal structure and biogenesis
A	0	0.0	RNA processing and modification
K	124	3.6	Transcription
L	146	4.3	Replication, recombination and repair
B	3	0.0	Chromatin structure and dynamics
D	29	0.8	Cell cycle control, mitosis and meiosis
Y	0	0.0	Nuclear structure
V	30	0.8	Defense mechanisms
T	129	3.7	Signal transduction mechanisms
M	85	2.5	Cell wall/membrane biogenesis
N	47	1.3	Cell motility
Z	0	0.0	Cytoskeleton
W	0	0.0	Extracellular structures
U	26	0.7	Intracellular trafficking and secretion
O	98	2.8	Posttranslational modification, protein turnover, chaperones
C	142	4.1	Energy production and conversion
G	124	3.6	Carbohydrate transport and metabolism
E	205	6.0	Amino acid transport and metabolism
F	66	1.9	Nucleotide transport and metabolism
H	125	3.6	Coenzyme transport and metabolism
I	65	1.9	Lipid transport and metabolism
P	137	4.0	Inorganic ion transport and metabolism
Q	38	1.1	Secondary metabolites biosynthesis, transport and catabolism
R	374	10.9	General function prediction only
S	213	8.2	Function unknown
-	1281	37.5	Not in COGs
